# Tracheotomy in Laryngeal Diphtheria

**Published:** 1885-09

**Authors:** 


*Tracheotomy in Laryngeal Diphtheria.* By R. W. PARKER. Pp. 124. Second Edition. London: H. K. LEWIS. 1885.

This is an excellent little work, giving in clear, simple language a thoroughly reliable account of the most approved methods of treating laryngeal diphtheria. The general principle underlying the treatment advocated is "that as a largely local disease, diphtheria at all stages requires local treatment." This principle is further amplified in certain suggestive "aphorisms:"

"The local condition in diphtheria precedes the general; the latter depends largely on the absorption of the septic products of the local condition.

"The disease spreads locally by the contagion of its own products.

"Spread of the disease must be combated by the complete destruction and removal of the products of the local lesion.

"Death very generally occurs from extension of the disease into the lungs.

"The value of any form of treatment may be gauged by the success with which extension of the disease is prevented.

"The operation of tracheotomy is only indicated where there is evidence of mechanical obstruction in the larynx or trachea.

"Retraction of the soft parts of the anterior chest wall during inspiration, together with the more or less complete suppression of voice, are the indications *par excellence* for the performance of tracheotomy.

"Tracheotomy is in no sense a therapeutic measure; therefore, neither the local nor the general treatment of diphtheria should be suspended after the performance of the operation.

"The chief objects of the operation are to facilitate treatment, and to guard against death from mechanical causes while this treatment is being carried out.

"To be of service, the operation must be performed while the disease is local, and before its spread towards the lungs or general infection of the system places the patient beyond the reach of surgical help."

The purely practical part of the work is particularly well handled. We are impressed with the possible advantages of the author's angular tube, and mean to give it a trial. We heartily commend the book to our readers.

